# Opposition behaviour against the third wave of autocratisation: Hungary and Poland compared

**DOI:** 10.1057/s41304-021-00325-x

**Published:** 2021-04-21

**Authors:** Gabriella Ilonszki, Agnieszka Dudzińska

**Affiliations:** 1 Department of Political Science, Corvinus University of Budapest, 8 Fővám tér, Budapest, 1093 Hungary; 2 Institute of Sociology, University of Warsaw, Krakowskie Przedmieście 26/28, 00-927 Warsaw, Poland

**Keywords:** Autocratisation, Extra-parliamentary arena, Hungary, Opposition, Parliament, Party system, Poland

## Abstract

Hungary and Poland are often placed in the same analytical framework from the period of their ‘negotiated revolutions’ to their autocratic turn. This article aims to look behind this apparent similarity focusing on opposition behaviour. The analysis demonstrates that the executive–parliament power structure, the vigour of the extra-parliamentary actors, and the opposition party frame have the strongest influence on opposition behaviour, and they provide the sources of difference between the two country cases: in Hungary an enforced power game and in Poland a political game constrain opposition opportunities and opposition strategic behaviour.

## Introduction

### What can this study add?

Hungary and Poland are often packed together in political analyses on the grounds that they constitute cases of democratic decline. The parties in governments appear infamous on the international, particularly on the EU, scene. Fidesz^1^ in Hungary has been on the verge of leaving or being forced to leave the People’s Party group due to repeated abuses of democratic norms, and PiS in Poland^2^ is a member of the European Reformists and Conservatives EU party group. Both regimes represent cases of democratic decline (Maerz et al. 2020). The life of opposition is rarely easy as executives tend to dominate the parliamentary agenda, and particularly difficult when the governing forces represent a new political–strategic rationale as opposed to their antecedents.

**Fig. 1:**
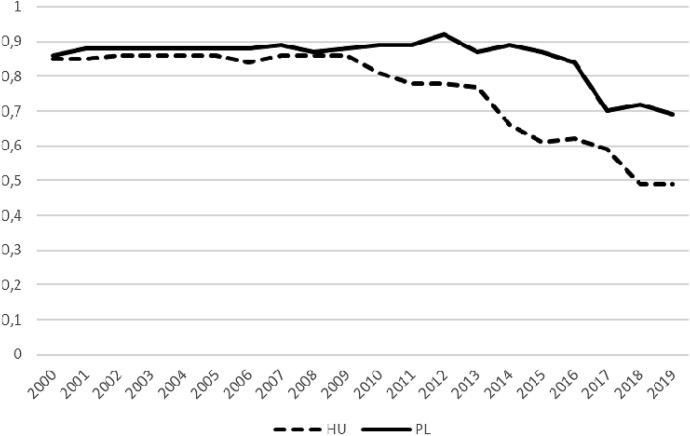
Electoral democracy index (EDI) for Hungary and Poland 2000–2019. *Data source*: Coppedge et al. (2020)

At first glance, the similarity of the political processes in Hungary and Poland is well grounded in data; both in Hungary and in Poland the process of autocratisation is taking place (Lührmann and Lindberg 2019). The curves of the electoral democracy index (EDI)^3^ look quite similar: in Hungary EDI began to decline in 2010 and in Poland in 2015 when Fidesz and PiS came to power, respectively (Fig. 1). It is therefore reasonable to suppose that the deterioration of indicators is related to new governments in these countries. In fact, this marks the starting point for our comparative analysis.

The article aims to add to existing research in three respects. First, by comparing the opposition behaviour in the two countries we provide a dynamic picture that helps explain the causes, the adaptation processes as well as the differences and similarities of opposition behaviour. Our expectation is that despite the apparent similarity between the two cases the patterns of government–opposition relations are far from identical (Bustikova and Guasti 2017). Second, by exceeding broad generalisations that prevail regarding the two countries that are too often placed in the same box we shall explore substantive features of the two cases focusing on the behaviour of opposition.^4^ Lastly, as most opposition studies on non-democracies tend to focus on long-existing non-democratic regimes (Gandhi and Przeworski 2006; Loidolt and Mecham 2016; Gandhi et al. 2020) Hungary and Poland offer an important complementary perspective as they had experienced apparently successful democratisation with a well-working government–opposition power structure. Specifically, we enquire whether the opposition in these two post-communist countries has similar or different functions than in long-standing established autocracies?

## Opposition in context—varied expectations

Both Fidesz and PiS experienced eight years in parliament as main opposition parties when they acquired government power. In Hungary, our analysis will cover the 2010–2020 decade, i.e. the three Orbán governments (2010–2014, 2014–2018, and the ongoing 2018–term). Fidesz, together with its small satellite party ally (Christian Democrats, KDNP), enjoys a two-thirds supermajority in parliament, allowing them uninhibited constitutional and legislative action—which, indeed, they have widely used. These governments are usually regarded as coalitions. The status of the minor party is, however, questionable: they run an electoral alliance with Fidesz and form a party alliance with them (even dual-party membership is allowed). However, in parliament both parties operate independent parliamentary party groups (PPGs), but mainly to secure a maximum of parliamentary resources. In political and policy terms, the KDNP is hardly visible.

For Poland, the time frame of the analysis is shorter. The 2015 PiS government was the first one after 1989 that enjoyed a one-party majority and faced a president from the same party. However, under the formal one-party label, PiS hosted two other small parties: the radical–conservative Solidarity Poland (SP) and liberal–conservative Porozumienie. These parties campaigned on ‘one-party’ lists and later formed a ‘one-party’ PPG. After the parliamentary election in 2019, the one-party majority of PiS has been retained, albeit with more intra-party competition as both satellite parties increased their share. At the same 2019 parliamentary elections, PiS lost their majority in the Senate, which marked an unprecedented change as both chambers used to have identical political composition before. Although the Senate is a weak institution, it has 30 days to take a position on a bill, which is an important asset in times of exceptionally accelerated legislation. Also, the Speaker of the Senate (now from the opposition) enjoys a major public standing.

**Fig. 2:**
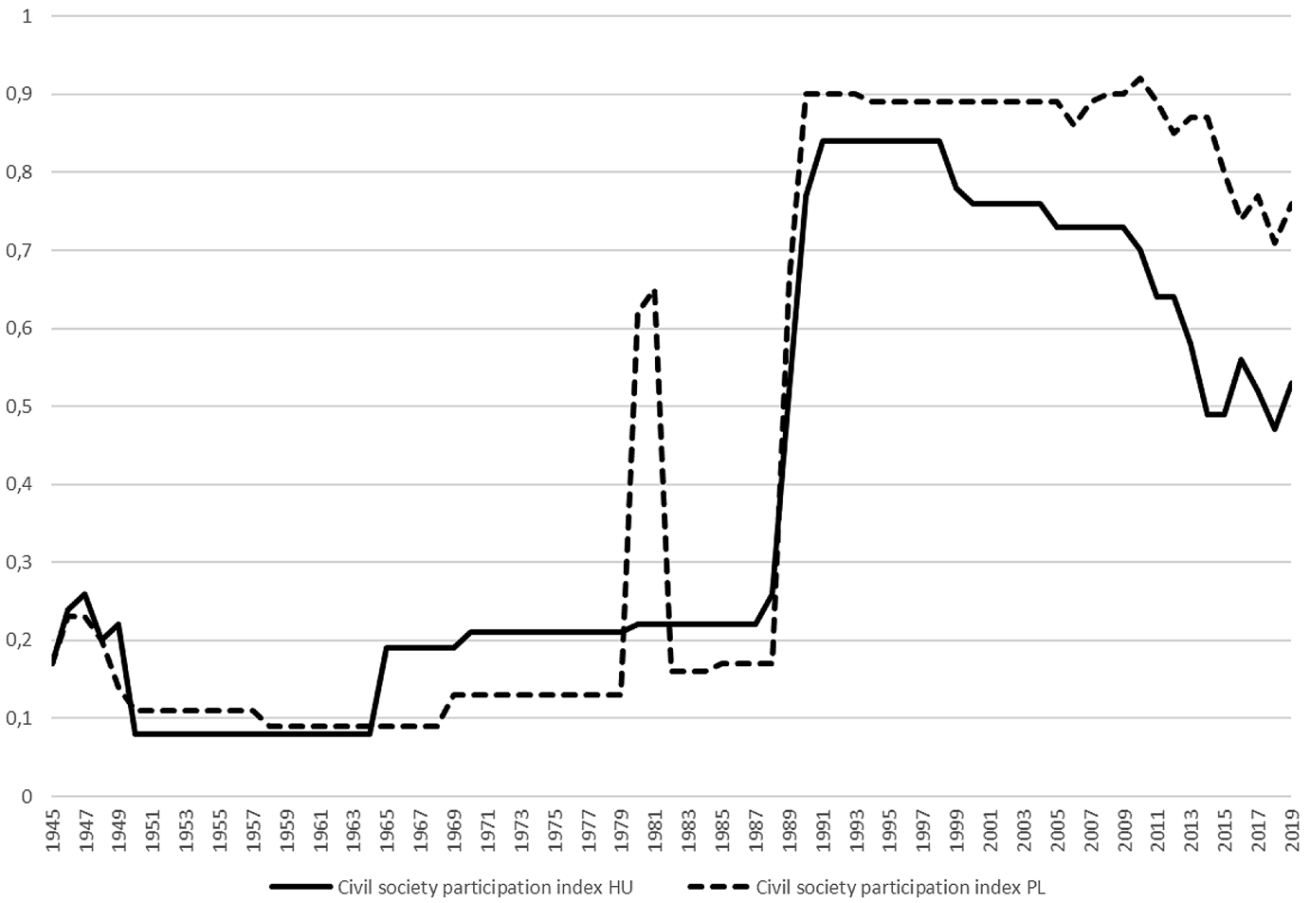
Civil society participation in Hungary and in Poland 1945–2019. *Data source*: Coppedge et al. (2020)

In addition to the government–parliament focus, two further aspects are paramount for the government–opposition power structure, civil society and the media. In Hungary, civil society actors tend to be sceptical towards parties, party–civil society cooperation remains sporadic, and until 2018 parties were not welcome at civil society demonstrations. Moreover, as early as 2017, the executive introduced legislation against civil society actors in Hungary. By contrast, in Poland a vivid civil society prevails; citizen response to political events is common and not limited to Warsaw. Political parties join street protests, using them as a non-parliamentary instrument of opposition to the government. The Civil Society Participation Index (Fig. 2) shows that during the period of communism civil society in Hungary was even stronger than in Poland, but this changed after the establishment of the Solidarity trade union, and then after a short reversal has persisted ever since.

In Hungary, free public media do not exist. According to estimations, the Fidesz media empire constitutes 77.8% of the media market (Mérték Médiaelemző Műhely 2019). In Poland, public broadcasters are politically controlled by the parliamentary majority, but the private sector is much stronger and mostly explicitly liberal. Although since 2015 PiS has been repeatedly announcing the ‘re-polonisation’ of the private media by forcing the inclusion of a larger share of Polish capital or by market deconcentration, nothing like this happened (Palczewski 2018) until December 2020 when a state-owned company bought a local media group from a German owner. The group controls 20 out of 24 regional titles and 120 local newspapers, along with over 17 million monthly users of their websites (Shotter 2020). Even earlier, after 2015, when laws related to the prevention of terrorist attacks came into force, new provisions have allowed for greater control of content on the Internet. Moreover, both in Poland and Hungary journalists tend to face limitations regarding access to and working in parliament. Overall, though, freedom of expression and access to alternative sources of information, as well as civil society, are still in a reasonably good condition in Poland—certainly much more so than in Hungary. According to the data from some recent research conducted at the University of Warsaw,^5^ freedom of speech was valued the fourth highest out of 22 evaluated policy fields (6.9 on an 11-point scale from 0 = state works badly to 10 = state works well). These different civic and media environments form part of the larger political opportunity structure of the opposition.

Table 1 shows a more complete comparative overview of the most important variables that can shape opposition behaviour. Some of these factors are systemic (constitutional), such as the political position of the president, the structure of the parliament or the electoral system, while others may change over time and thus constitute a temporary configuration, such as party complexion and format of the government or media control by the government.

**Table 1:** The institutional context

	Hungary	Poland
President	Weak (elected by the parliament, veto to be overridden by a simple majority)	Strong (elected in popular vote, veto to be overridden by 2/3 majority)
Parliament	Unicameral	Bicameral: Senate (the upper house) with modest influence on legislation
Electoral system	Mixed (106 FPTP majority, one-seat districts; 93 PR, one national district)	PR (Sejm, 41 districts); FPTP majority (Senate, 100 one-seat districts)
Local governments	Weak	Strong
Rules of the parliamentary standing orders	Difficult to change (with 2/3 majority)	Easy to change (with simple majority)
Coalition government	Yes, formally—the dominant Fidesz with a small subordinate satellite party	Yes, not formally—the dominant PiS with two small satellite parties that since 2019 become more independent due to their increased seat share
Format of government	Surplus majority	Simple majority
Share of seats for ruling parties	67%	51% in lower chamber (a minority of 49% in upper chamber)
State-controlled media	78%	16% public radio, 26% public television
Civil society	Weak	Strong

In the following sections, we focus on both parliamentary and extra-parliamentary opposition behaviour. Given the differences between our two countries regarding the power of civil society and the connection between high politics and low politics’ actors, we expect to find variation in frequency, strength and the impact of extra-parliamentary opposition action. There is a growing understanding in the international literature that opposition cannot be equated with parliamentary party opposition only. Given the differences between Hungary and Poland in terms of the power of civil society, the question remains whether the historical difference persists in hard times when the democratic credentials of the system are under threat? The potentially persistent or changing inter-country difference is related to the intra-country developments: will the tendency towards autocratisation change the interaction between the parliamentary and extra-parliamentary opposition?

Moreover, we expect behavioural differences due to the differences in the composition of opposition in two respects, one regarding the structure of the opposition and the other regarding the ideological–political position of individual opposition parties. As to the first, Table 2 shows the share of opposition parties by party family in the two countries. For Hungary the three figures in each cell indicate that the observation period covers three parliamentary terms, while for Poland the two figures show that the observation period is two terms. The annotation shows the party composition of the respective party families.

**Table 2:** Share of opposition party families during the observation periods (at the beginning of each term)

Party family/country	Share of opposition (%)				
	*Left*	*Green*	*Liberal*	*Agrarian*	*Right*
Hungary	15.3; 14.6; 17.3	4.1; 2.5; 4.5	–	–	12.2; 11.6; 13.1
Poland	0; 10.7	0; 0	36.1; 29.1	3.5; 6.5	9.1, 2.4

Hungary—*left*: 2010, and 2014 MSzP (Socialists), 2018: MSzP, DK (Democratic Coalition), P (Dialogue); *green*: LMP (Politics Can Be Different); *radical right*: Jobbik (For a Better Hungary)

Poland—*left* 2019: Left (coalition of Together, Democratic Left Alliance and Spring), *green*: 2019 the Green Party run from joint lists with PO and belongs to KO PPG, *liberal*: 2015 PO (Civic Platform), N (Modern), 2019: KO (Civic Coalition: PO, N, Polish Initiative, Greens), *agrarian* 2015: PSL (Polish Peasants Party), 2019: Polish Coalition (PSL, Kukiz’15), *right*: 2015 Kukiz15, 2019 Konfederacja (Confederation)

A mere glimpse at Table 2 reveals that the Hungarian opposition scene is more polarised, making any potential opposition cooperation difficult. Moreover, due to the absence of a dominant party among them, opposition parties would be more competitive striving for the ‘top’ position. The Polish opposition scene is considerably more diverse and more flexible as compared to the apparently rigid Hungarian one. It is also skewed to the left-liberal side. Still, importantly, all opposition PPGs in the Sejm share their criticism of PiS policy and constitute an alternative (party or coalition of parties) to the populist government, for which there is no equivalent in Hungary. In addition to the structure of the opposition, the individual parties’ political–ideological positions might also play a role in their behaviour as these will influence the strategies of the individual parties. For example, it might seem a rewarding and attractive strategy for some parties to cooperate with or at least offer some cooperation to the governing party in the hope of future posts. For others, either due to their former political or current ideological position this is not an option. Thus, opposition parties are invariably committed to overcoming the regime.

## The changing behaviour of the opposition

In both countries, the opposition faced powerful government legislative action from the very beginning. In Hungary, the two-thirds majority and in Poland the unprecedented institutional concentration of one-party power were important triggers in this regard. The Sejm was like a voting machine (Dudzińska 2016). In Hungary, the number of bills accepted in 2010–2014 was more than twice as high as during the first democratic term (1990–1994) when the process of systemic change had to be managed (Ilonszki and Vajda 2021). However, anti-government parties may use different strategies. In Poland, three months after the election the PO’s leader declared: ‘We will be the total opposition, the hardest possible’ and the party boycotted negotiations with the ruling party (Szczerbiak 2017), while the liberal Modern declared several times it would be open to discuss matters with PiS. The differences between opposition parties were pronounced in Hungary—at least in the first years of the populist government. First, Jobbik tried to build up cooperative strategies and initiated policies that also fit the governing party’s political and policy views. Nevertheless, Fidesz did not need Jobbik as it had a safe majority position in parliament. Moreover, Fidesz did not want to replace its ‘invisible’ coalition partner KDNP with Jobbik, which had its own political and policy identity. Besides, Fidesz and Jobbik targeted the same constituency; thus, they appeared to be rivals on the electoral scene. Within just a few years, Jobbik has made a strategic turn towards the centre and the opposition parties became fully united in an anti-government stance.

In both countries, the opposition was active both at the level of legislation and parliamentary scrutiny. In Poland, due to the more flexible party framework joint legislative initiatives were common (i.e. around seven percent), while in Hungary there was just one single joint opposition legislative initiative—not surprisingly given the polarised opposition scene—during the first parliamentary term (2010–2014), that between Socialists (MSzP) and the Greens (LMP). In a way this increased legislative activity is paradoxical as opposition legislative initiatives are virtually never accepted in Hungary (Várnagy and Ilonszki 2018). In contrast, 5.5% of opposition bills were enacted in Poland in 2015–2019.^6^ The former governing party PO in Poland has shown less intense activity than the other opposition groups—confirming the academic knowledge that senior opposition parties that expect to return to the government benches challenge the government either with legislative action or scrutiny less frequently than smaller or permanent opposition parties (De Giorgi and Ilonszki 2018: 236). This, however, does not hold for the Socialists in Hungary, who had been the senior governing party and the rival of Fidesz. The difference can be explained by the relatively strong position of the Civic Platform as a challenger in Poland, while the Hungarian MSzP gets ailing. We can also observe major differences in using scrutiny measures. For example, interpellations are used much more frequently in Poland than in Hungary. In Poland interpellations are structurally encouraged by the preferential list vote system, that puts individual MPs more into focus, while in Hungary they are discouraged by the possibility of abusing interpellations by the government parties.^7^ This shows the power of extra-parliamentary (electoral system) and intra-parliamentary regulations (standing orders) at the same time.

Clearly, the differences in standing rules, and how they are used or abused, also explain opposition action. In Hungary, the standing rules must be accepted by a two-thirds majority. Paradoxically, the two-thirds requirement was introduced at the democratic transition as a safety measure in defence of minority—now it serves supermajority interests. A series of standing order reforms substantially transformed parliamentary procedures in the past decade. This included the curtailing of opposition and speaking rights; the rules to establish investigation committees (once being the right of one-fifth of MPs now being made dependent on the will of the parliamentary majority); and an increase in the disciplinary powers of the Speaker. Such institutional changes alone do not tell the whole story, though. From a political perspective, the intention is more obvious: the governing party prepares for the long term—should they lose their two-thirds majority their predominant positions in parliament will remain. While in Hungary the standing rules have been substantially modified to the detriment of the opposition, in Poland they have remained largely unchanged, although they actually can be changed by a simple majority. The amendments of 2018 that gave the Marshal (Speaker) the right to consider whether a deputy’s statement or behaviour would ‘violate the seriousness’ of the Sejm or violate peace and order in the area managed by the Chancellery of the Sejm and to raise financial penalties on that basis, marked the only significant changes since 2015.

The disciplinary powers of the Speaker, such as financial penalty against MPs or their exclusion from parliamentary sessions, increased considerably in Hungary as well. While in Poland one single case was publicised, in Hungary financial penalty against MPs was applied in 22 cases between 2014 and 2018 and in 45 cases between 2018 and 2020. MPs were excluded from parliamentary sessions in three cases in the 2014–2018 parliamentary term and in eight cases since 2018 as a disciplinary measure. These disciplinary regulations in Hungary have not halted opposition action. Rather, they have elicited public response in the form of private donations from citizens to cover penalties. Difference between opposition parties in terms of ‘undisciplined’ behaviour has also prevailed: small parties, and individual MPs without a party or PPG background are less disciplined. It is difficult to judge the consequences: they have gained public visibility and media coverage—at least in the remaining very few independent media—while they have remained disconnected to democratic parties. This seems to have changed, as of lately: as a fully united opposition strategy is being formed for the 2022 parliamentary elections, these independent MPs might become precious assets for the more ‘disciplined’ opposition parties.

Despite the apparently different government trajectories, the pressure on the opposition in both countries is obvious in at least two important respects: at the level of the legislative process and regarding disciplinary measures. Although these pressures have been much harsher in Hungary than in Poland, fully fledged opposition action has emerged quicker in Poland than in Hungary—both in parliament and in the street.

In Poland, the opposition turned to civil society early on. Social movements and organisations began to protest immediately after the first legislative decisions of PiS. The leaders of PiS labelled new opposition’s activities outside the parliament ‘ulica i zagranica’ (‘streets and abroad’), words that ironically referred to a speech of a communist party leader from 1968 addressing activities of the anti-communist opposition. The strongest street protests, supported by the opposition, concerned three laws that would affect the court system. Social pressure prompted the president to veto two of them. Two other contested projects related to abortion, which sparked a wave of feminist and leftist demonstrations. As a result, neither of these laws was enacted.

In addition, the activities of the opposition in the Sejm extended beyond legislation and ‘normal’ parliamentary work. They frequently resorted to filibuster (e.g. prolongation of parliamentary debates by multiple speakers or hundreds of amendments) or occupied the pulpit, especially in the first years. A radical novelty was that the opposition occupied the plenary hall from 16 December 2016 to 12 January 2017, in reaction to an attempt to change the organisation of the work of journalists in the Sejm and the exclusion of an opposition MP from the plenary. Street protests supported the parliamentary opposition. Both the former rules of journalists’ work in the Sejm and the MP’s participation in the plenary were restored.

The space and place of the dynamics of opposition action was different in Hungary. Initially, the parliamentary opposition was overwhelmed by the parliamentary activities, and the first large civic movements for the freedom of media and for freedom of education did not expect and did not aim for any party support. It became obvious, however, by the second term that the classic parliamentary tools would be insufficient for the opposition (Várnagy and Ilonszki 2018). The conflicts culminated in 2018, when the government introduced a new labour law. In parliament, the opposition parties tried to filibuster and boycott the vote, and on the street different civic groups and political parties organised joint demonstrations. This was the first occasion that Jobbik and Socialist sympathisers were marching together in Budapest listening to speakers from highly different backgrounds. These demonstrations spread to other cities as well. Opposition parties increasingly sought to find extra-parliamentary ways in face of the frail parliamentary opportunities. There has been increasing cooperation between the opposition groups in parliament favoured by a decreasing level of party polarisation (for example, opposition parties initiated several investigation committees together, though with rather limited impact).

Learning cooperative strategies eventually brought about some opposition success both in Hungary and Poland. In Poland, at the 2018 local elections PO and Modern created the Civic Coalition to increase their chances. Later, informal coalitions at the 2019 parliamentary elections and common candidates in each of the 100 FPTP Senate districts improved the status of the opposition. In Hungary, at the 2019 local elections Budapest and several other major cities fell to the opposition—also based on joint opposition efforts or/and the inclusion of civic actors.

The new power structure facilitated opposition to take advantage of more institutionalised and thus more effective forms of activity instead of violent protests in Poland, while in Hungary conflicts further intensified. Two examples might be illustrative: In Poland, MPs with the help of their mandate can and effectively do acquire information and thus exercise control over state administration, local governments and state-owned companies. In contrast, in Hungary when opposition party MPs wanted to read out their proclamation in the public television and thus inform the people that public television does not provide reliable information, they were thrown out of the building and some beaten up by security guards. The Public Prosecutor claimed that security guards only performed their duty. The case now is at the European Court of Justice.

In Poland, it is also possible for the opposition to use direct presidential elections as a lever for success in parliamentary elections, provided there is a favourable sequence of terms. PiS took this opportunity when it came to power in 2015, largely thanks to the result of the presidential election a few months earlier.

The context of the pandemic only confirms the former analysis. In Hungary, the regime became harsher both in parliament and outside. The crisis provided arguments to implement steps against local governments where the opposition acquired majority position. The introduction of decree governing raised serious concerns given that the government implemented this without a sunset clause. Also in Poland, the COVID-19 pandemic has confirmed the need for a strong state and thus strengthened the government. PiS wanted to capitalise on this by sticking to the scheduled presidential election day in May 2020. Ultimately, they failed. The joint efforts including the Senate, local governments, NGOs, media and even one of the intra-PiS satellites,^8^ eventually inhibited the presidential election to be held under changed and non-transparent rules and conditions. Finally, the Left organised a roundtable on the presidential election with the participation of all PPGs to agree on a new compromise law so that it could be smoothly passed in parliament (Polish Press Agency 2020). This was an exceptional meeting in recent years.

## Conclusions

Our starting point was that in our two countries different opportunity structures and constraints would likely give rise to different opposition behaviour. This has been broadly confirmed by our analysis. The executive in Poland was unable to pursue some of its proposed steps against the opposition ‘infrastructure’, like the full transformation of the media or the court system, due to its less stable position as opposed to Hungary where the executive plays the ‘game of attack on the entire field’—to quote the Prime Minister, a devoted football fan. Some of the opposition’s behavioural differences cannot be explained by mere institutional variation, however. Rather, institutional opportunities and the opposition party framework as well as the individual party strategies all combine in shaping opposition behaviour in the two countries.

The number of actors and their cooperation potential can be identified as important explanatory factors in this regard. Thus, while it is reasonable to conclude that government action affects opposition action, the connection is not necessarily immediate or straightforward. Blondel’s argument (Blondel 1997) on the parasitic nature of opposition can be applied to our cases with some qualification: the impact of the government on opposition behaviour has been largely shaped by the composition of the opposition party frame and features of civil society. Rigid party lines, a higher level of party polarisation and the presence of genuinely new parties have inhibited or at least delayed opposition moves against excessive executive action in Hungary.

As for the differential engagement of civil society, it is not only their historical differences that matter but also more recent patterns of party and civil society relations. There have been striking time-related differences in the otherwise conspicuously parallel dynamics: fast reaction of the opposition in Poland and slow adaptation to the context in Hungary. Nevertheless, the closer a country proceeds on the line of autocratisation, the more the role of non-parliamentary opposition is on the increase: in Hungary, the increasing rigidity of government forces has melted the icy connections between intra-parliamentary and extra-parliamentary opposition.

Despite these substantial differences, we have also identified some similar trajectories of opposition behaviour. In both countries, the opposition has used extra-parliamentary means (although more frequently and more systematically in Poland than in Hungary); they increasingly have resorted to more robust intra-parliamentary activities, such as boycott and filibuster; and not least they developed common strategies for concerted oppositional action.

The analysis of opposition behaviour has also helped us to detect important aspects of the two regimes. After 1989, both countries adapted democracy as a political system and capitalism as an economic system. Their transformation largely followed the model of Western liberal democracy. The analysis of a longer trend of indicators may suggest that we are facing a downward correction of the liberal component. Nevertheless, the recent experiences in Poland demonstrate that several opportunities are available for the opposition to constrain the abuse of power by government. One can reasonably expect that the recent EDI decline could be stopped or reduced. In contrast, the opposition opportunities and democratic revival remain bleak in Hungary.

When it comes to opposition opportunities, our conclusion is different for the two countries. As specified in the introductory essay of the Symposium, ‘the strategic goal of governments in electoral autocracies cannot be reasonably reduced to simply banning any political oppositions altogether’ (see Helms in this issue). Indeed, in other non-democratic cases even dictators make concessions to the opposition in terms of policies, even though, ultimately, the outcome of the concessions is arbitrary (Gandhi and Przeworski 2006). Thus, the opposition gets some benefit and might enjoy some rent seeking in an authoritarian legislature above and exceeding its genuine interest, namely to maintain its visibility for the next round of elections. This seems to be a possible trend in Poland, where the opposition not only ‘demonstrates’ the presence of multiparty democracy, but also serves the government as a convenient whipping boy who can be blamed for any government errors. In addition, both sides, government and opposition, seem to profit from extreme political polarisation. By contrast, we cannot see anything like this in Hungary. The opposition is extremely vulnerable and kept only for legitimacy reasons.

In any regime, the actual implementation of the ideal of polyarchy, including free access to information, freedom of speech and freedom of association, depends both on the strength of governments and—much more so—on the strength of oppositions. Again major differences between our two cases stand out: in Poland, we witness a *political game* between government and opposition—even, if arguably this game is more brutal than normal for established democracies. In contrast, in Hungary we witness an *enforced power game* dominated by the executive leaving hardly any terrain for opposition action.

## Footnotes

1 The party’s full name now reads Fidesz—Hungarian Civic Alliance.

2 Abbreviation of Prawo i Sprawiedliwość (Law and Justice).

3 See Coppedge et al. (2020). The EDI runs on a continuous scale from 0 to 1, with higher values indicating a more democratic dispensation. We operationalise autocratisation as a substantial decline on the EDI (within one year or over a connected time period). A decline is substantial if it amounts to drop of 0.1 or more on the EDI. A typical example would be the series of declines in democratic qualities in Hungary from 2006 to 2017 adding up to drop of the EDI of 0.11.

4 A rare and welcome exception is Csehi and Zugt (2020). They do not only argue for the presence of differences in the two countries’ Eurosceptic populism but the government–opposition power structure is one of the explanatory factors in their analysis, as well.

5 Ongoing project ‘Political system in action’, led by A. Dudzińska, unpublished research.

6 Official data of the Chancellery of Sejm.

7 In Hungary, governing parties use interpellations excessively, and their interpellations get publicity on the floor in higher share than opposition interpellations (Ilonszki and Vajda 2021).

8 Porozumienie at least temporarily turned into a veto player. Gowin, then deputy Prime Minister, publicly questioned the feasibility of holding the election on 10 May as planned by PiS and resigned from his position in government. Five MPs from the party voted against or abstained.
